# Folate Levels in Patients Hospitalized with Coronavirus Disease 2019

**DOI:** 10.3390/nu13030812

**Published:** 2021-03-02

**Authors:** Eshcar Meisel, Orly Efros, Jonathan Bleier, Tal Beit Halevi, Gad Segal, Galia Rahav, Avshalom Leibowitz, Ehud Grossman

**Affiliations:** 1 Department of Internal Medicine “D”, Sheba Medical Center, Tel Hashomer, Ramat Gan 52621, Israel; orlyefros@gmail.com (O.E.); jobleier@gmail.com (J.B.); talbh142@gmail.com (T.B.H.); Avshalom.Leibowitz@sheba.health.gov.il (A.L.); 2 Sackler Faculty of Medicine, Tel Aviv University, Tel Aviv 6997801, Israel; Gad.Segal@sheba.health.gov.il (G.S.); Galia.Rahav@sheba.health.gov.il (G.R.); Ehud.Grossman@sheba.health.gov.il (E.G.); 3 National Hemophilia Center and Institute of Thrombosis & Hemostasis, Sheba Medical Center, Tel Hashomer, Ramat Gan 52621, Israel; 4 Department of Internal Medicine “T”, Sheba Medical Center, Tel Hashomer, Ramat Gan 52621, Israel; 5 Infectious Disease Unit, Sheba Medical Center, Tel Hashomer, Ramat Gan 52621, Israel; 6 Internal Medicine Wing, Sheba Medical Center, Tel Hashomer, Ramat Gan 52621, Israel

**Keywords:** folic acid, folic acid deficiency, folate, folate deficiency, COVID-19, SARS CoV-2, epidemic, hypoxemia, invasive ventilation

## Abstract

We aimed to investigate the prevalence of decreased folate levels in patients hospitalized with Coronavirus Disease 2019 (COVID-19) and evaluate their outcome and the prognostic signifi-cance associated with its different levels. In this retrospective cohort study, data were obtained from the electronic medical records at the Sheba Medical Center. Folic acid levels were available in 333 out of 1020 consecutive patients diagnosed with COVID-19 infection hospitalized from January 2020 to November 2020. Thirty-eight (11.4%) of the 333 patients comprising the present study population had low folate levels. No significant difference was found in the incidence of acute kidney injury, hypoxemia, invasive ventilation, length of hospital stay, and mortality be-tween patients with decreased and normal-range folate levels. When sub-dividing the study population according to quartiles of folate levels, similar findings were observed. In conclusion, decreased serum folate levels are common among hospitalized patients with COVID-19, but there was no association between serum folate levels and clinical outcomes. Due to the important role of folate in cell metabolism and the potential pathologic impact when deficient, a follow-up of folate levels or possible supplementation should be encouraged in hospitalized COVID-19 patients. Fur-ther studies are required to assess the prevalence and consequences of folate deficiency in COVID-19 patients.

## 1. Introduction

Folate, also known as vitamin B9, is an essential water-soluble micronutrient that plays a key role in the nucleic acid synthesis and normal cellular function. Mammals cannot synthesize folate and must absorb it from the diet, with serum folate levels decreasing within several days of dietary folate restriction [1,2,3]. Although a single measurement of decreased folate level is insufficient to determine chronic folate deficiency, and measurements of homocysteine levels or red blood cells (RBC) folate are usually required, plasma folate measurement provides an appropriate assessment of general folate intake [2,4,5].

Folate deficiency is associated with several diseases in the adult population, including megaloblastic anemia, cardiovascular disease, colon cancer, neuropathy, depression, hypercoagulability, and cognitive decline [6]. Decreased folate levels were also reported to be associated with several viral and bacterial infections, such as influenza, parvovirus, Epstein-Barr virus, mycoplasma pneumonia [7], and lower respiratory tract infections in young children [8]. A possible biological explanation may include the important role of folate in supporting the innate and adaptive immune system by maintaining natural killing (NK) cell cytotoxic activity, T-helper 1 (Th1) mediated immune response, and antibody production [9,10,11].

The Coronavirus Disease 2019 (COVID-19) pandemic emerged in December 2019, resulting in a tremendous global death toll and a major worldwide economic burden. Its clinical presentation varies from fever, cough, and other non-specific symptoms, to pulmonary involvement that may lead to respiratory failure and death [12,13,14,15]. Severe COVID-19 can also involve other systems such as the cardiovascular, coagulation, and renal systems [12,14,15]. Several studies have proposed a link between folate levels and COVID-19 prevalence, with a possible association to the disease severity [16]. Acosta-Elias et al. suggested that folic acid supplementation has a protective potential, leading to lower COVID-19 hospitalization rates in pregnant women [17]. Im et al., in a recent study including 50 patients, showed that COVID-19 infection was not associated with folate deficiency [18]. Moreover, the main carrier responsible for transporting folate into intestinal cells following folate digestion is the proton-coupled folate transporter (PCFT). This high-affinity facilitative folate carrier is found mostly in the proximal jejunum and duodenum. PCFT appears to be the primary transporter of folate and folic acid into cells of the small intestine. Gene expression for PCFT is influenced, among other transcription factors, by vitamin D [1,19]. As vitamin D deficiency was associated with a higher infection rate and poor outcomes in COVID-19 patients [20,21,22,23,24], we hypothesized that folate levels in COVID-19 patients would be decreased.

In this study, we aimed to evaluate folate levels in a large cohort of hospitalized patients with COVID-19 and investigate its prognostic significance.

## 2. Methods

### 2.1. Study Design and Population

This is a retrospective cohort study. Data were obtained from electronic medical records at the Sheba Medical Center, the largest tertiary medical center operating in Israel. We retrieved all consecutive patients hospitalized from 27 January 2020, to 23 November 2020, with a diagnosis of COVID-19 confirmed by PCR. All patients included were 18 years old or older at the time of diagnosis and had at least one serum folate level measurement during hospitalization. If there was more than one folate measurement during hospitalization, the lower level was chosen.

### 2.2. Data Collection and Outcomes

For each patient, baseline demographic characteristics and clinical information were extracted from the medical records. Clinical data included medical comorbidities, chronic medications, vital signs on admission (body temperature, systolic blood pressure, oxygen saturation, body mass index (BMI)), and laboratory tests. Preexisting comorbidities were defined based on the International Classification of Disease, 10th Revision codes. They included heart failure, ischemic heart disease, chronic kidney disease, malignancy, chronic obstructive pulmonary disease, diabetes mellitus, hypertension, cerebrovascular accident, and dyslipidemia. If death occurred outside the hospital within 60 days of index admission, mortality dates were obtained by matching patient data with the Israeli national population registry.

The primary outcomes were mortality and a composite outcome of invasive ventilation and mortality. Additional secondary outcomes included: Death within 60 days of hospitalization; intubation within 30 days from admission, including only patients who were intubated during the initial hospitalization with COVID-19; length of hospital stay; hypoxemia during hospitalization, defined as a measurement of oxygen saturation <90% using pulse oximetry; acute kidney injury (AKI), defined according to the Kidney Disease: Improving Global Outcomes (KDIGO) definition as a ratio of more than 1.5 between the maximal creatinine level to its minimal value in less than seven days [25].

The study was conducted and reported in accordance with the Strengthening the Reporting of Observational Studies in Epidemiology (STROBE) reporting guidelines [26].

Serum folate levels were measured in a Beckman-Coulter DxI 800 chemiluminescent immunoassay. According to the laboratory reference range, decreased levels of folate were defined as lower than 5.9 ng/mL (13.37 nmol/L). Serum folate quartiles were also calculated and used for data analysis.

The study was approved by the institutional review board of the Sheba Medical Center.

### 2.3. Statistical Analysis

Baseline clinical data were compared between patients with decreased serum folate levels and normal-range serum folate levels. Continuous variables were compared using the Mann–Whitney–Wilcoxon test, and categorical variables were compared using the Chi-square test.

Logistic regression was applied to identify best-associated variables with a low serum folate level following COVID-19 diagnosis. Covariates for the multivariable models were pre-specified based on clinical relevance.

Outcomes of mortality, invasive ventilatory support, length of hospital stay, and AKI were analyzed in the following manner. The crude association between low serum folate levels and mortality and low serum folate levels and the composite outcome of invasive ventilation and mortality was modeled using Kaplan–Meier curves and compared using the Log Rank test. A Cox proportional hazard regression analysis was performed to adjust for age, sex, BMI, background comorbidities (atrial fibrillation, ischemic heart disease, heart failure, hypertension, diabetes mellitus, chronic kidney disease), chronic medications (angiotensin-converting enzyme, angiotensin II receptor blockers, beta-blockers, and statins), low systolic blood pressure at admission (defined as <90 mm of mercury (mmHg)), and maximal creatinine level as measured up to 24-h from admission.

Data analyses were performed using the R programming language (R Development Core Team, version 3.6.2, Vienna, Austria).

## 3. Results

### 3.1. Baseline Characteristics

From January 27th, 2020, to November 23rd, 2020, a total of 1060 adult patients were hospitalized with a polymerase chain reaction (PCR) confirmed diagnosis of COVID-19 infection at the Sheba Medical Center. Of these, 333 patients (31.4%) were tested for folate levels during their hospitalization. Among these patients, 38 (11.4%) had decreased folate levels. A total of 23 patients (6.9%) were treated with folic acid supplements. Of those receiving supplementation, 5 (13.2%) had low folate levels, and 18 (6.1%) had normal-range folate levels. Baseline characteristics of patients with decreased measured folate levels vs. normal-range folate levels are presented in Table 1. The prevalence of atrial fibrillation and chronic kidney disease (CKD) was significantly higher in the group of patients with decreased folate levels. There was no significant difference in the hemoglobin level and mean cell volume (MCV) between the groups. Patients’ baseline characteristics are presented in Table 1.

In order to further examine the effect of quantitative folate levels on COVID-19 disease, we subdivided the study population according to quartiles of folate levels. Patients’ baseline characteristics according to quartiles are presented in Table 2. Patients with folate levels at the third and fourth quartiles had significantly higher vitamin B12 levels compared to the patients with lower median serum folate levels. There was not a significant difference in the hemoglobin level between the groups.

### 3.2. Predictors for Decreased Folate Levels in Patients with COVID-19

In a multivariable analysis, patients with atrial fibrillation had an increased risk of having decreased folate levels during hospitalization (odds ratio (OR) = 3.93; 95% confidence interval (CI); *p*-value = 0.022). Patient sex, age, and other inspected comorbidities were not associated with an increased probability of having decreased folate levels. The complete data of the predictors for decreased folate levels in COVID-19 patients are presented in Table 3.

### 3.3. Patient Outcome

Out of the 333 patients enrolled in our study, 20 patients developed acute kidney injury. A total of 76 patients required invasive ventilation within 30 days of admission, and 65 patients had died within 60 days of admission. No significant difference was found in the incidence of inspected outcomes (acute kidney injury during hospitalization, hypoxemia, a need for invasive ventilation within 30 days from admission, mortality within 60 days of hospitalization, and length of hospital stay) between hospitalized COVID-19 patients with normal-range folate levels and decreased folate levels (Table 4). Similar results were seen when hospitalized COVID 19 patients were grouped according to folate level quartiles, with no significant difference in outcomes between groups. The data regarding outcomes of patients hospitalized with COVID-19 stratified by serum folate levels are presented in Table 5.

Cox proportional hazard regression analysis for the association of mortality and a composite outcome of intubation and mortality, adjusted for folate levels, patients’ comorbidities, low systolic blood pressure at presentation, creatinine level at presentation, and treatment with folic acid, are presented in Table 6 and Table 7. Decreased folate levels were not found to be associated with an increased mortality risk or a composite outcome of intubation and mortality risk.

**Figure 1:**
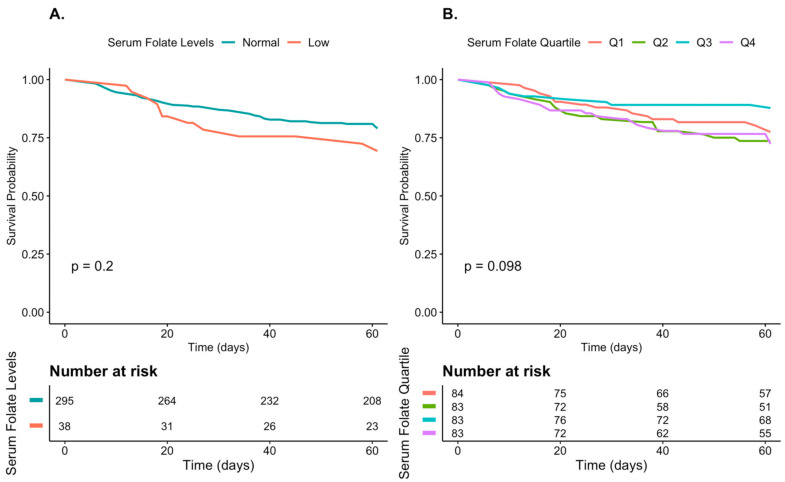
Kaplan-Meier survival curve in patients hospitalized with COVID-19. A. For normal-range and low folate levels B. For quartiles of the patients’ folate levels. No significant difference in the survival rate was observed between patients with decreased and those with normal-range folate levels (**A**) or according to quartiles of folate levels (**B**).

**Figure 2:**
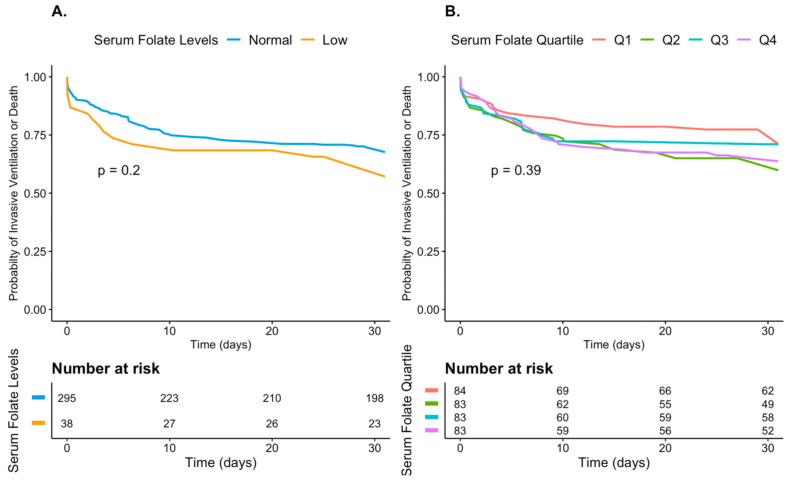
Accumulative probability plot for a composite outcome of invasive ventilatory support and mortality in patients hospitalized with COVID-19 A. For normal-range and low folate levels B. For quartiles of the patients’ folate levels. No significant difference in the probability for a composite outcome was observed between patients with decreased and those with normal-range folate levels (**A**) or according to quartiles of folate levels (**B**).

A Kaplan–Meier plot stratified by folate levels is shown in Figure 1A,B. Figure 2A,B demonstrates the cumulative incidence of the composite outcome of invasive ventilation and mortality. No significant difference in the survival rate (Figure 1) and in the probability for a composite outcome (Figure 2) was observed between patients with decreased and those with normal-range folate levels (A) or according to quartiles of folate levels (B). When examining the effect of quantitative folate levels on hospital stay length, need for intubation, and mortality, no specific trend was observed (supplementary Figures S1 and S2 and Tables S1 and S2).

## 4. Discussion

This study evaluated the frequency of decreased folate levels among patients hospitalized with COVID-19 and investigated its prognostic implications. We also subdivided the study population into quartiles according to serum folate levels to better examine whether an increase in serum folate levels affects clinical outcomes. We found a high frequency (11.4%) of decreased folate levels in patients hospitalized with COVID-19. There was no difference in clinical outcome that included mortality or a composite outcome of invasive ventilation and mortality in patients with decreased folate levels hospitalized with COVID-19. Similar results were seen when comparing patients according to quartiles of folate levels.

Folate is an essential micronutrient that is crucial for one-carbon metabolism. It facilitates the transfer of a one-carbon unit in the purine and pyrimidine synthesis, metabolism of methionine, serine, and glycine, and the formation of methylating agents required for metabolism and gene regulation. Thus, when folate levels are insufficient, the cell’s ability to remethylate homocysteine is impaired, resulting in higher levels of plasma homocysteine [3,5]. Folate status is most often assessed by measuring folate concentrations in the plasma, serum, or red blood cells. As serum or plasma folate levels may reflect recent dietary intake, the diagnosis of true folate deficiency must be followed by repeated measurements. Serum folate concentrations below 3 ng/mL are suggestive of folate deficiency. Other laboratory tests such as elevated homocysteine levels and decreased red blood cell folate concentrations, which better reflect the folate tissue status, may aid in the diagnosis of folate deficiency [1,2,4,5]. To note, for population surveys, serum and plasma folate measurements provide an appropriate assessment of general folate status [5]. In this study, we used serum folate levels as red blood cells folate and homocysteine levels are not routinely measured. Decreased folate levels were referred when at least one laboratory measurement during hospitalization was below 5.9 ng/mL (13.37 nmol/L), according to the laboratory immunoassay reference range used at the Sheba Medical Center. Therefore, we could not draw conclusions regarding folate deficiency in our study population. Nevertheless, in the past years, numerous studies have shown a marked decrease in folate deficiency prevalence in Western populations, with similar trends observed among hospitalized patients [27]. These trends are attributed mainly to national implementation of product fortification with folic acid [28,29]. In our study, we found a higher-than-expected decrease in serum folate levels without association to disease outcomes. This observation merits further studies of folate status in COVID-19 patients.

When comparing the background comorbidities between patients hospitalized with COVID-19 with decreased or with normal-range folate levels, the former group had a higher prevalence of chronic kidney disease and atrial fibrillation. Previous small observational studies reported similar results [30,31]. A possible explanation may be inadequate dietary intake in these patients [32]. This assumption is further supported by the higher vitamin B12 levels observed in patients in the higher median of folate levels (third and fourth quartiles).

One main characteristic of folate deficiency is megaloblastic macrocytic anemia [6]. In our study, there was no significant difference in hemoglobin levels and MCV between the decreased folate group and the normal-range group and between the folate quartile groups. This may also emphasize the short-term decrease in folate levels as reflected by serum folate level measurements, which does not necessarily indicate a folate-deficient state. Nevertheless, this short-term decrease may have clinical relevance in the long term if left untreated.

As previously discussed, folate plays an important role in supporting the innate and adaptive immune system, with poor folate status shown in previous studies to be a risk factor for viral and bacterial infections, including the lower respiratory tract [7,8]. Recent studies suggested a possible association between folic acid and SARS-CoV−2. An inhibitory interaction between folic acid and the proteolytic protein furin, which was documented to promote activation of the coronavirus, was recently demonstrated [33,34]. Additionally, folic acid and its derivatives were found to have a direct anti-SARS-CoV−2 effect [35,36]. Singh et al. proposed the incorporation of folic acid in the treatment regimen of COVID-19 [37]. Moreover, Wiltshire et al. suggested that high doses of folic acid may have a beneficial effect on hypoxemic COVID-19 patients’ pulmonary perfusion by its activity on endothelial nitric oxide and nitric oxide availability [38].

Another interesting aspect to note is the biological contribution of vitamin D to folate absorption. As noted earlier, vitamin D functions as a transcription factor of the proton-coupled folate transporter (PCFT) gene, thus increasing its expression. PCFT protein is an essential component in folate absorption as it is responsible for folate transport into intestinal cells [1,19]. Indeed, folate levels were demonstrated to be positively correlated with vitamin D levels in several studies [39,40]. To date, much has been published regarding a possible association between vitamin D deficiency, COVID-19 infection, and disease severity [20,21,22,23,24,41]. Moreover, Annweiler et al. demonstrated that vitamin D supplementation was associated with a higher survival rate and lower COVID-19 disease severity [42]. Thus, as vitamin D is proposed to be deficient in COVID-19 infected patients with severe disease, lower observed folate levels would be possibly expected in these patients. Katz et al. showed that vitamin D deficient patients were more likely to be infected with COVID-19 [20]. Accordingly, decreased folate levels in COVID-19 hospitalized patients, regardless of disease severity, would be expected. This is consistent with our findings. In the current study, vitamin D levels were not routinely measured, thus direct correlation could not be assessed.

## 5. Study Limitations

This study has several limitations. The studied population was small, as folate levels were not widely measured. Folate was routinely measured in patients hospitalized with COVID-19 only during several periods throughout the pandemic, leading to a potential selection bias. Furthermore, the patients were not routinely tested for RBC folate and homocysteine levels, thus we could not determine a folate-deficient state.

Acute kidney injury was defined according to the KDIGO definition of a ratio of more than 1.5 between the maximal and minimal creatinine levels at a seven-day period. Urine output and 48-h elevation were not considered, as well as changes in creatinine values that were non-minimal or non-maximal. Thus, there is an underestimation of AKI (acute kidney injury) events.

## 6. Study Conclusions

To date, this is the largest study investigating serum folate levels in patients hospitalized with COVID-19. We have shown that decreased folate levels are highly prevalent in these patients. The folate levels were not found to be associated with disease severity and prognosis. This study highlights the importance of evaluating folate levels in COVID-19 patients during hospitalization and providing supplementation accordingly to prevent a potential future folate-deficient state in these patients. Further studies are required to examine folate deficiency rates and their clinical importance in these patients.

## Figures and Tables

**Table 1:** Patients’ baseline characteristics.

Baseline Characteristics	Decreased Folate Levels (*n* = 38)	Normal Folate Levels (*n* = 295)	*p*-Value
Sex [Male, *n* (%)]	29 (76.3)	189 (64.1)	0.189
Age (years) (median [IQR ^d^])	64.90 [53.93, 73.55]	64.61 [51.53, 74.35]	0.868
Body mass index (Kg/m^2^). (median [IQR])	27.70 [23.45, 30.85]	27.00 [24.13, 30.50]	0.74
Temperature ^a^- Celsius (median [IQR])	37.80 [37.02, 38.60]	37.90 [37.30, 38.50]	0.641
Oxygen saturation ^a^	94.98 (4.46)	94.27 (7.06)	0.403
Pulse rate ^a^–beats per minute	85.28 (16.02)	83.84 (15.36)	0.605
Systolic blood pressure ^a^- mmHg ^b^ (median [IQR])	110.00 [93.25, 122.00]	106.00 [94.50, 119.50]	0.563
Hypertension- *n* (%)	20 (52.6)	129 (43.7)	0.387
Ischemic heart disease- *n* (%)	7 (18.4)	42 (14.2)	0.659
Diabetes mellitus- *n* (%)	16 (42.1)	85 (28.8)	0.136
Cerebrovascular accident- *n* (%)	4 (10.5)	24 (8.1)	0.85
Heart failure- *n* (%)	6 (15.8)	36 (12.2)	0.714
Malignancy- *n* (%)	5 (13.2)	48 (16.3)	0.796
Atrial fibrillation- *n* (%)	9 (23.7)	23 (7.8)	0.005
Chronic obstructive pulmonary disease- *n* (%)	1 (2.6)	12 (4.1)	1.00
Chronic kidney disease- *n* (%)	13 (34.2)	37 (12.5)	0.001
Chronic anemia- *n* (%)	11 (28.9)	51 (17.3)	0.129
Dyslipidemia- *n* (%)	15 (39.5)	99 (33.6)	0.588
B12 therapy- *n* (%)	0 (0.0)	9 (3.1)	0.575
Iron therapy- *n* (%)	1 (2.6)	6 (2.0)	1.00
Folic acid therapy- *n* (%)	5 (13.2)	18 (6.1)	0.202
D-dimer ^a^- nmol/L (median [IQR])	5.57 [2.77, 9.16]	5.28 [3.17, 9.67]	0.816
Hemoglobin ^a^- g/L (median [IQR])	126.1 [108.9, 142.4]	129.0 [114.0, 141.1]	0.776
C-reactive protein ^a^- mg/L (median [IQR])	88.30 [29.90, 148.28]	85.24 [27.20, 158.80]	0.98
Troponin I ^a^- µg/L (median [IQR])	0.110 [0.00585, 0.05520]	0.01040 [0.00568, 0.02560]	0.518
Creatinine ^a^- µmol/L (median [IQR])	91.94 [71.60, 172.38]	84.86 [69.84, 107.85]	0.039
Platelets ^a^- 10^9^/L (median [IQR])	189.50 [139.25, 252.75]	193.50 [145.75, 267.25]	0.736
International normalized ratio ^a^- *n* (median [IQR])	1.07 [1.02, 1.29]	1.11 [1.03, 1.22]	0.678
Albumin ^a^- g/L (median [IQR])	35.0 [31.0, 38.0]	37.0 [33.0, 40.0]	0.049
Creatine kinase ^a^- µkat/L (median [IQR])	2.24 [1.34, 4.22]	1.9 [1.09, 3.82]	0.565
Vitamin B12 ^a^- pmol/L (median [IQR])	0.3 [0.23, 0.46]	0.3 [0.19, 0.47]	0.561
Ferritin ^a^- µg/L (median [IQR ^d^])	481.80 [221.88, 821.52]	344.00 [154.30, 713.50]	0.172
Iron ^a^- µmol/L (median [IQR])	6.09 [3.76, 9.93]	5.37 [3.45, 8.23]	0.487
Lactate dehydrogenase ^a^ µkat/L (median [IQR])	5.6 [4.44, 8.54]	6.16 [4.53, 7.84]	0.747
White blood cells ^a^- 10^9^/L (median [IQR])	6.33 [5.07, 8.72]	6.42 [4.87, 9.83]	0.82
Red blood cells ^a^-10^12^/L (median [IQR])	4.53 [4.08, 4.93]	4.65 [4.06, 5.00]	0.548
Mean cell volume- fL (median [IQR])	86.77 [83.10, 89.38]	87.38 [82.92, 90.62]	0.634
Macrocytosis ^c^- *n* (%)	0 (0.0)	3 (1.2)	1.00

^a^ Measured within 24-h from admission. ^b^ Millimeter of mercury. ^c^ Mean cell volume above 100fL.d Interquartile range.

**Table 2:** Patient baseline characteristics according to folate level quartiles.

	Q1 (Folate <7.4)	Q2 (Folate 7.4−10.4)	Q3 (Folate 10.4–14.8)	Q4 (Folate >14.8)	*p*-Value
*n*	84	83	83	83	
Sex [Male, *n* (%)]	55 (65.5)	63 (75.9)	55 (66.3)	45 (54.2)	0.034
Age (years) (median [IQR ^d^])	62.32 [51.17, 73.41]	64.57 [53.21, 73.11]	59.57 [48.94, 72.75]	68.49 [57.49, 77.72]	0.038
BMI (Kg/h^2^). (median [IQR])	26.70 [24.22, 30.03]	27.30 [24.08, 30.60]	27.10 [23.70, 30.50]	27.00 [23.40, 30.63]	0.993
Temperature ^a^- Celsius (median [IQR])	37.50 [37.08, 38.60]	38.10 [37.45, 38.50]	37.90 [37.30, 38.50]	38.00 [37.20, 38.60]	0.18
Oxygen saturation ^a^(median [IQR])	96.00 [95.00, 98.00]	95.00 [93.00, 98.00]	96.00 [95.00, 98.00]	96.00 [93.00, 97.75]	0.271
Pulse rate ^a^- beats per minute (median [IQR])	84.00 [75.00, 94.00]	85.00 [77.50, 95.00]	81.00 [70.00, 95.00]	85.00 [74.50, 93.50]	0.194
Systolic blood pressure ^a^- mmHg ^b^ (median [IQR])	107.00 [95.00, 120.00]	106.00 [92.00, 118.00]	105.00 [92.50, 117.00]	106.00 [96.00, 121.50]	0.792
Hypertension- *n* (%)	41 (48.8)	40 (48.2)	33 (39.8)	35 (42.2)	0.569
Ischemic heart disease- *n* (%)	12 (14.3)	9 (10.8)	11 (13.3)	17 (20.5)	0.341
Diabetes mellitus- *n* (%)	33 (39.3)	19 (22.9)	21 (25.3)	28 (33.7)	0.078
Cerebrovascular accident- *n* (%)	6 (7.1)	4 (4.8)	10 (12.0)	8 (9.6)	0.369
Heart failure- *n* (%)	9 (10.7)	7 (8.4)	10 (12.0)	16 (19.3)	0.175
Malignancy- *n* (%)	15 (17.9)	15 (18.1)	12 (14.5)	11 (13.3)	0.778
Atrial fibrillation- *n* (%)	11 (13.1)	6 (7.2)	7 (8.4)	8 (9.6)	0.604
Chronic obstructive pulmonary disease- *n* (%)	2 (2.4)	3 (3.6)	4 (4.8)	4 (4.8)	0.823
Chronic kidney disease- *n* (%)	16 (19.0)	10 (12.0)	7 (8.4)	17 (20.5)	0.094
Chronic anemia- *n* (%)	17 (20.2)	16 (19.3)	7 (8.4)	22 (26.5)	0.026
Dyslipidemia- *n* (%)	31 (36.9)	26 (31.3)	29 (34.9)	28 (33.7)	0.895
B12 therapy- *n* (%)	1 (1.2)	5 (6.0)	0 (0.0)	3 (3.6)	0.079
Iron therapy- *n* (%)	1 (1.2)	3 (3.6)	1 (1.2)	2 (2.4)	0.654
Folic acid therapy- *n* (%)	6 (7.1)	7 (8.4)	3 (3.6)	7 (8.4)	0.571
D-dimer ^a^- nmol/L (median [IQR])	5.57 [3.05, 10.39]	5.94 [3.19, 19.63]	4.26 [3.00, 6.66]	5.28 [3.03, 8.81]	0.117
Hemoglobin ^a^- g/L (median [IQR])	125.4 [110.0, 141.0]	129.0 [109.5, 143.8]	131.7 [120.7, 141.4]	127.7 [111.0, 139.0]	0.316
C-reactive protein ^a^- mg/L (median [IQR])	91.94 [30.99, 157.58]	95.00 [34.72, 180.60]	69.97 [13.26, 148.97]	71.69 [26.41, 145.01]	0.172
Troponin I ^a^- µg/L (median [IQR])	0.00960 [0.00550, 0.02370]	0.01165 [0.00565, 0.02850]	0.00985 [0.00630, 0.02117]	0.01090 [0.00570, 0.03147]	0.985
Creatinine ^a^- mg/dL (median [IQR])	87.52 [65.42, 109.62]	86.63 [73.37, 108.73]	83.10 [69.84, 108.73]	87.52 [67.18, 112.27]	0.899
Platelets ^a^-10^9^/L (median [IQR])	216.50 [142.75, 269.25]	192.50 [150.50, 248.00]	192.00 [145.75, 260.75]	186.00 [138.25, 268.00]	0.581
International normalized ratio ^a^- *n* (median [IQR])	1.11 [1.06, 1.26]	1.12 [1.03, 1.22]	1.12 [1.00, 1.18]	1.11 [1.05, 1.22]	0.401
Albumin ^a^- g/L (median [IQR])	35.5 [31.0, 38.0]	36.0 [32.0, 39.0]	38.0 [34.0, 41.0]	36.5 [34.0, 40.7]	0.009
Creatine kinase ^a^- µkat/L (median [IQR])	1.95 [1.07, 3.99]	2.04 [1.35, 5.09]	1.77 [1.11, 3.35]	1.69 [0.95, 3.77]	0.289
Vitamin B12 ^a^- pmol/L (median [IQR])	0.28 [0.18, 0.46]	0.26 [0.14, 0.39]	0.31 [0.21, 0.48]	0.36 [0.23, 0.62]	0.015
Ferritin ^a^- µg/L (median [IQR])	359.60 [147.90, 572.20]	361.30 [197.80, 913.00]	388.20 [154.80, 808.20]	309.10 [171.48, 613.85]	0.526
Iron ^a^- µmol/L (median [IQR])	5.28 [3.58, 9.53]	5.28 [3.4, 7.61]	6.98 [4.3, 9.84]	4.39 [3.22, 6.22]	0.057
Lactate dehydrogenase ^a^- µkat/L (median [IQR])	5.74 [4.31, 7.72]	6.48 [4.67, 8.17]	6.29 [4.59, 8.5]	5.59 [2.17, 7.57]	0.153
White blood cells ^a^- 10^9^/L (median [IQR])	6.58 [5.45, 9.51]	6.90 [4.97, 10.12]	6.51 [4.43, 9.67]	5.54 [4.65, 9.04]	0.253
Red blood cells ^a^-10^12^/L (median [IQR])	4.64 [4.02, 5.02]	4.68 [4.05, 5.05]	4.62 [4.21, 4.89]	4.49 [3.98, 4.99]	0.819
MCV ^c^ (median [IQR])	87.40 [84.31, 90.12]	87.42 [82.42, 89.72]	86.88 [83.41, 90.69]	87.46 [82.25, 90.08]	0.770

^a^ Measured within 24-h from admission. ^b^ Millimeter of mercury. ^c^ Mean cell volume [MCV] above 100fL.^d^ Interquartile range.

**Table 3:** Predictors for decreased folate levels in the presence of COVID-19 diagnosis.

Characteristics	Odds Ratio (95% CI ^a^)	*p*-Value
Male sex	1.54 (0.61, 3.87)	0.359
Age	0.99 (0.96, 1.02)	0.46
Body Mass Index	0.95 (0.88, 1.03)	0.235
Atrial fibrillation	3.93 (1.22, 12.72)	0.022
Ischemic heart disease	0.72 (0.21, 2.42)	0.593
Heart failure	0.8 (0.21, 3.05)	0.749
Chronic kidney disease	2.43 (0.84, 7.02)	0.099
Diabetes mellitus	2.01 (0.77, 5.2)	0.152
Cerebrovascular accident	1.56 (0.41, 5.86)	0.513
Hypertension	1 (0.38, 2.66)	0.993
Malignancy	0.54 (0.16, 1.87)	0.33
Chronic anemia	1.61 (0.59, 4.36)	0.349
Chronic obstructive pulmonary disease	0.31 (0.03, 3.44)	0.34
C-reactive protein	1 (1, 1.01)	0.435

^a^ Confidence Interval.

**Table 4:** Outcomes of COVID-19 hospitalized patients.

Characteristics	Decreased Folate Levels (*n* = 38)	Normal Folate Levels (*n* = 295)	*p*-Value
Acute kidney injury- *n* (%)	1 (2.6)	19 (6.4)	0.57
Length of hospital stay- days (median [IQR ^a^])	7.00 [3.00, 11.00]	7.00 [4.00, 14.00]	0.727
Hypoxemia- *n* (%)	24 (63.2)	161 (54.6)	0.407
Invasive ventilation ^b^- *n* (%)	12 (31.6)	64 (21.7)	0.246
Mortality ^c^- *n* (%)	10 (30.3)	55 (20.9)	0.315

^a^ Interquartile range. ^b^ Within 30 days from admission. ^c^ Within 60 days of hospitalization.

**Table 5:** Outcome of COVID-19 hospitalized patients according to quartiles of folate levels.

Characteristics	Q1 (*n* = 84)	Q2 (*n* = 83)	Q3 (*n* = 83)	Q4 (*n* = 83)	*p*-Value
Acute kidney injury- *n* (%)	4 (4.8)	5 (6.0)	4 (4.8)	7 (8.4)	0.728
Length of hospital stay- days (median [IQR ^a^])	7.00 [4.00, 11.00]	8.00 [3.00, 14.00]	7.00 [4.00, 15.00]	8.00 [3.50, 14.00]	0.874
Hypoxemia- *n* (%)	47 (56.0)	50 (60.2)	43 (51.8)	45 (54.2)	0.735
Invasive ventilation ^b^- *n* (%)	16 (19.0)	20 (24.1)	20 (24.1)	20 (24.1)	0.823
Mortality ^c^- *n* (%)	16 (21.9)	21 (29.2)	9 (11.7)	19 (25.7)	0.057

^a^ Interquartile range. ^b^ Within 30 days from admission. ^c^ Within 60 days of hospitalization.

**Table 6:** Adjusted analysis for the association between decreased folate levels and mortality and a composite of invasive ventilation support and mortality in patients hospitalized with COVID-19.

	Mortality		Composite Outcome of Invasive Ventilation Support and Mortality	
Characteristics	Odds Ratio (95% CI ^a^)	*p*-Value	Odds Ratio (95% CI ^a^)	*p*-Value
Decreased folic acid levels	1.18 [0.57, 2.46]	0.656	1.67 [0.92, 3.05]	0.092
Atrial fibrillation	1.29 [0.64, 2.62]	0.475	1.07 [0.58, 1.96]	0.835
Ischemic heart disease	1.47 [0.80, 2.69]	0.209	1.07 [0.63, 1.82]	0.793
Heart failure	1.33 [0.65, 2.72]	0.431	1.62 [0.89, 2.93]	0.111
Chronic kidney disease	1.01 [0.48, 2.12]	0.978	0.82 [0.43, 1.55]	0.533
Diabetes mellitus	1.43 [0.81, 2.53]	0.218	1.26 [0.79, 2.01]	0.327
Cerebrovascular accident	0.63 [0.23, 1.73]	0.375	1.33 [0.65, 2.72]	0.433
Hypertension	1.28 [0.73, 2.25]	0.387	1.09 [0.70, 1.68]	0.707
Malignancy	2.43 [1.37, 4.30]	0.002	1.51 [0.92, 2.48]	0.103
Chronic anemia	1.48 [0.82, 2.69]	0.192	1.10 [0.66, 1.83]	0.707
Chronic obstructive pulmonary disease	0.62 [0.17, 2.25]	0.47	0.59 [0.21, 1.69]	0.325
Systolic blood pressure < 90 mmHg	2.02 [1.13, 3.64]	0.018	3.52 [2.25, 5.51]	<0.001
Creatinine	1.06 [0.90, 1.24]	0.486	0.98 [0.85, 1.12]	0.728
Folic acid therapy	0.73 [0.26, 2.04]	0.542	0.94 [0.43, 2.07]	0.884

^a^ Confidence Interval.

**Table 7:** Adjusted analysis for the association between quartiles of folate levels and mortality and a composite of invasive ventilation support and mortality in patients hospitalized with COVID-19.

	Mortality		Composite Outcome of Invasive Ventilation Support and Mortality	
Characteristics	Odds Ratio (95% CI ^a^)	*p*-Value	Odds Ratio (95% CI ^a^)	*p*-Value
Quartiles of Serum Folate levels	1.02 [0.82, 1.27]	0.854	1.02 [0.85, 1.22]	0.834
Atrial fibrillation	1.34 [0.67, 2.67]	0.402	1.22 [0.69, 2.18]	0.495
Ischemic heart disease	1.47 [0.80, 2.68]	0.213	1.06 [0.62, 1.79]	0.836
Heart failure	1.30 [0.64, 2.67]	0.471	1.48 [0.81, 2.69]	0.199
Chronic kidney disease	1.06 [0.51, 2.17]	0.883	0.92 [0.49, 1.72]	0.788
Diabetes mellitus	1.43 [0.81, 2.52]	0.223	1.28 [0.81, 2.04]	0.293
Cerebrovascular accident	0.64 [0.23, 1.75]	0.38	1.27 [0.62, 2.61]	0.512
Hypertension	1.30 [0.74, 2.28]	0.365	1.11 [0.72, 1.71]	0.644
Malignancy	2.43 [1.37, 4.31]	0.002	1.46 [0.89, 2.39]	0.137
Chronic anemia	1.50 [0.83, 2.71]	0.178	1.13 [0.68, 1.86]	0.648
Chronic obstructive pulmonary disease	0.57 [0.16, 2.10]	0.402	0.55 [0.19, 1.60]	0.273
Systolic blood pressure <90 mmHg	2.01 [1.12, 3.61]	0.02	3.38 [2.17, 5.28]	<0.001
Creatinine	1.06 [0.91, 1.24]	0.447	0.98 [0.85, 1.13]	0.822
Folic acid therapy	0.73 [0.26, 2.05]	0.548	0.98 [0.44, 2.15]	0.956

^a^ Confidence Interval.

## Data Availability

Data is contained within the article or supplementary material.

## Supplementary Materials

The following are available online at https://www.mdpi.com/2072-6643/13/3/812/s1, Figure S1: Length of hospital stay as a function of folate levels, Figure S2: Quantitative folic acid levels (ng/mL) in patients who did not require intubation (0) and in patients who required intubation (1). Bottom Quantitative folic acid levels (ng/mL) in living patients within 60 days of hospitalization (0) and in patients who did not survive (1), Table S1: The baseline characteristics of patients that had measured folate levels and patients who did not, Table S2: The laboratory characteristics of patients that had measured folate levels and patients who did not.
