# Supplementary material for: Folate Levels in Patients Hospitalized with Coronavirus Disease 2019
Source: Nutrients. 2021 Mar 2;13(3):812. doi: 10.3390/nu13030812 (PMC8001221; doi:10.3390/nu13030812)
Supplement: Supplementary file 1 [file nutrients-13-00812-s001.zip › 1104180-suppl/Supplementary figure 2.pdf]

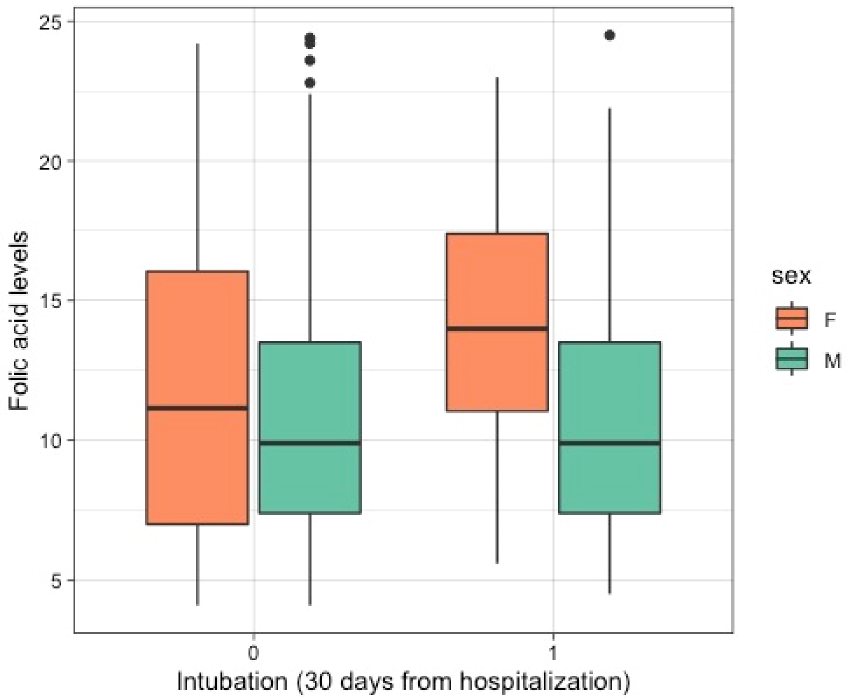

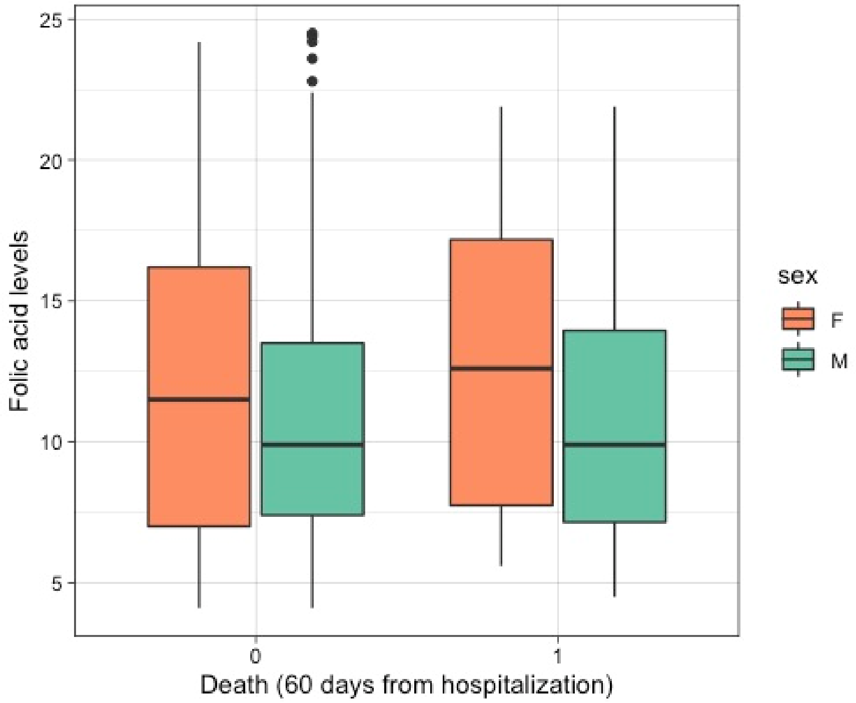

Supplementary figure 2. Top Quantitative folic acid levels (ng/ml) in patients who did not require intubation (0) and in patients who required intubation (1). **Bottom** Quantitative folic acid levels (ng/ml) in living patients within 60 days of hospitalization (0) and in patients who did not survive (1).
