# Supplementary material for: Folate Levels in Patients Hospitalized with Coronavirus Disease 2019
Source: Nutrients. 2021 Mar 2;13(3):812. doi: 10.3390/nu13030812 (PMC8001221; doi:10.3390/nu13030812)
Supplement: Supplementary file 1 [file nutrients-13-00812-s001.zip › 1104180-suppl/Table S1.pdf]

baseline characteristics	Folic Acid was Taken (n = 333)	Folic acid was not Taken (n = 727)	P-value
**Sex = male (%)**	218 (65.5)	378 (52.0)	<0.001
**Age- years (median [IQR])**	64.61 [51.65, 74.28]	64.61 [48.12, 78.73]	0.52
**BMI- n (median [IQR])**	27.00 [24.05, 30.50]	27.30 [24.20, 31.00]	0.597
**Temperature [a]- Celsius (median [IQR])**	37.90 [37.20, 38.60]	37.70 [37.10, 38.40]	0.015
**Systolic blood pressure [a]- mmHg [b] (median [IQR])**	106.00 [94.00, 120.00]	110.00 [100.00, 121.00]	0.003
**Hypertension- n (%)**	149 (44.7)	289 (39.8)	0.143
**Ischemic heart disease- n (%)**	49 (14.7)	75 (10.3)	0.049
**Diabetes mellitus- n (%)**	101 (30.3)	166 (22.8)	0.011
**Cerebrovascular accident- n (%)**	28 (8.4)	89 (12.2)	0.081
**Heart failure- n (%)**	42 (12.6)	55 (7.6)	0.011
**Malignancy- n (%)**	53 (15.9)	87 (12.0)	0.096
**Atrial fibrillation- n (%)**	32 (9.6)	75 (10.3)	0.807
**Chronic obstructive pulmonary disease- n (%)**	13 (3.9)	29 (4.0)	1
**Chronic kidney disease- n (%)**	50 (15.0)	65 (8.9)	0.004
**Chronic anemia- n (%)**	62 (18.6)	83 (11.4)	0.002
**Dyslipidemia- n (%)**	114 (34.2)	194 (26.7)	0.015
**B12 therapy- n (%)**	9 (2.7)	15 (2.1)	0.669
**Iron therapy- n (%)**	7 (2.1)	26 (3.6)	0.275
**Folic acid therapy- n (%)**	23 (6.9)	76 (10.5)	0.084

Supplementary table 1. [a] Measured within 24-hours from admission. [b] Millimeter of mercury.
