# Supplementary material for: Folate Levels in Patients Hospitalized with Coronavirus Disease 2019
Source: Nutrients. 2021 Mar 2;13(3):812. doi: 10.3390/nu13030812 (PMC8001221; doi:10.3390/nu13030812)
Supplement: Supplementary file 1 [file nutrients-13-00812-s001.zip › 1104180-suppl/Table S2.pdf]

**Laboratory characteristics**	**Folic Acid was Taken (n = 333)**	**Folic acid was not Taken (n = 727)**	**P-value**
**D-dimer [a]- ng/ml (median [IQR])**	972.50 [573.50, 1793.75]	875.00 [532.00, 1686.00]	0.549
**Hemoglobin [a]- g/dl (median [IQR])**	12.81 [11.20, 14.11]	12.98 [11.76, 14.20]	0.175
**C-reactive protein [a]- mg/l (median [IQR])**	86.97 [27.11, 158.71]	62.44 [17.41, 133.74]	0.006
**Troponin [a]- ng/l (median [IQR])**	10.40 [5.65, 26.45]	9.90 [5.30, 22.90]	0.326
**Creatinine [a]- mg/dl (median [IQR])**	0.98 [0.79, 1.24]	0.88 [0.70, 1.16]	<0.001
**Platelets [a]- K/microL (median [IQR])**	193.50 [144.00, 267.00]	188.00 [147.00, 247.00]	0.371
**International normalized ratio [a]- n (median [IQR])**	1.11 [1.03, 1.22]	1.09 [1.01, 1.19]	0.034
**Albumin [a]- g/dl (median [IQR])**	3.60 [3.30, 4.00]	3.70 [3.30, 4.00]	0.448
**Creatine kinase [a]- IU/l (median [IQR])**	114.00 [66.00, 231.00]	110.00 [64.00, 235.00]	0.602
**Folic acid [a]- ng/ml (median [IQR])**	10.40 [7.40, 14.80]	NA [NA, NA]	NA
**Vitamin B12 [a]- pq/ml (median [IQR])**	405.00 [265.00, 631.00]	474.00 [255.00, 724.00]	0.118
**Ferritin [a]- ng/ml (median [IQR])**	359.60 [156.30, 762.60]	313.40 [151.93, 691.20]	0.778
**Iron [a]- mcg/dl (median [IQR])**	30.00 [20.00, 47.00]	29.00 [21.00, 47.00]	0.9
**Lactic dehydrogenase [a]- IU/l (median [IQR])**	365.00 [269.50, 473.00]	321.00 [238.00, 445.00]	<0.001
**White blood cells [a]- K/microL (median [IQR])**	6.42 [4.95, 9.67]	6.73 [4.94, 9.37]	0.652
**Red blood cells [a]- M/microL (median [IQR])**	4.62 [4.06, 5.00]	4.66 [4.19, 5.09]	0.079
**Mean cell volume- fL (median [IQR])**	87.36 [82.93, 90.08]	86.55 [83.72, 90.01]	0.79
**Macrocytosis [b]- n (%)**	3 (1.1)	15 (2.4)	0.293

Supplementary table 2. [a] Measured within 24-hours from admission. [b] Mean cell volume [MCV] above 100fL.
