# Supplementary figures and images for: Folate Levels in Patients Hospitalized with Coronavirus Disease 2019
Source: Nutrients. 2021 Mar 2;13(3):812. doi: 10.3390/nu13030812 (PMC8001221; doi:10.3390/nu13030812)

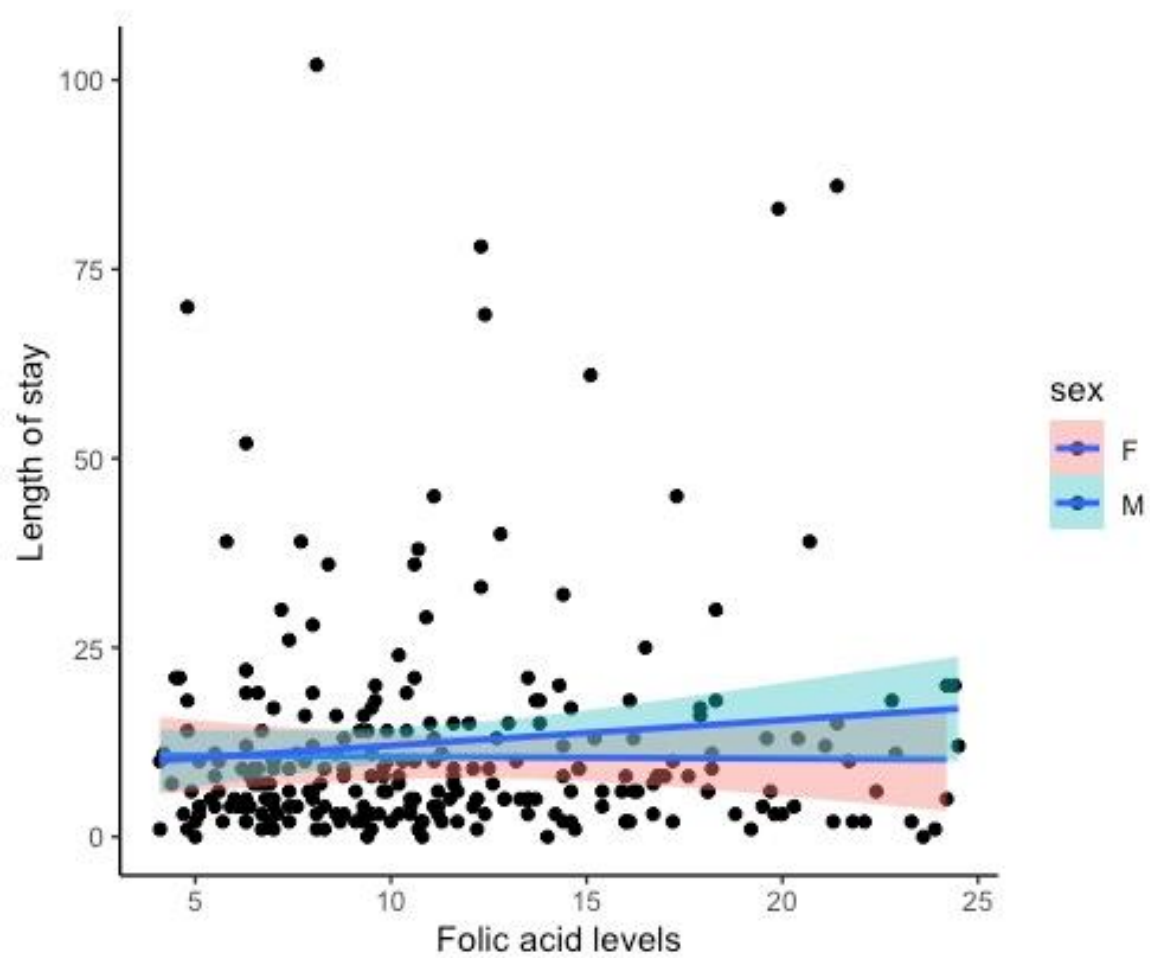

**Supplementary figure 1.** Length of hospital stay in days, as a function of folic acid levels.

Supplement: Supplementary file 1 [file nutrients-13-00812-s001.zip › 1104180-suppl/supplementary figure 1.pdf]
